# Decision to delivery interval, maternal and fetal outcomes in emergency caesarean sections in a tertiary teaching hospital, Dar es salaam, Tanzania

**DOI:** 10.4314/ahs.v23i3.5

**Published:** 2023-09

**Authors:** Peter Wangwe, Mfaume Kibwana, Furaha August, Aman I Kikula

**Affiliations:** 1 Department of Obstetrics and Gynaecology, Muhimbili University of Health and Allied Sciences, Muhimbili National Hospital, Obstetrics and Gynecology; 2 Geita Regional Referral Hospital, Obstetrics and Genecology

**Keywords:** Caesarean section category, maternal and fetal outcome, decision to delivery interval

## Abstract

**Background:**

Emergency caesarean section (CS) answers the question on how soon the procedure should be performed. Maternal and fetal outcomes deteriorate when decision to delivery interval (DDI) exceeds 75 min. This study aimed at determining the DDI, Maternal and fetal outcomes in CS categories at Muhimbili National Hospital (MNH).

**Methodology:**

A descriptive cross-sectional study involving 427 emergency CS at MNH was conducted from September to November, 2017. Data was extracted and analysed using SPSS version 23.0 where frequency, means, chi-square test and DDI were calculated to determine its association with categories of CS.

**Results:**

The mean DDI for category one, two and three CS were 126.73, 133.57 and 160.08 min respectively. Only two (0.5%) and 54 (12.6%) of category one and two emergency CS met the recommended DDI of 30 and 75 min respectively. Maternal and fetal adverse outcome were increasing with increase in DDI. There was no significant association between DDI and adverse maternal outcome (OR: 1.2; 95% CI 0.49-2.83) and fetal outcome (OR: 1.7; 95% CI 0.91-3.38).

**Conclusion:**

The proportions of adverse maternal and fetal outcome were high when DDI was ≥ 75 min. Improving triage of the patients according to their urgency is crucial in reducing prolonged DDI.

## Background

Classification of emergency caesarean section (CS) based on degree of urgency, answers the question of “when” (how quickly) the procedure should be performed. This requires the team (Midwives, Obstetrician and Anesthesiologist) to communicate on how to improve maternal and fetal outcomes. Basing on degree of urgency, caesarean sections is classified into four categories as per modified classification proposed by Lucas et al 1. Category one (emergency) caesarean sections (failed assisted or operative vaginal delivery with fetal distress, cord prolapse, placental abruption, placenta praevia with profuse bleeding or major antepartum haemorrhage and impending rupture of uterus) are performed when there is an immediate threat to the life of the woman or fetus. Category two (urgent) (failed assisted vaginal delivery without fetal distress, malpresentation in labour, previous caesarean deliveries in labour and failure to progress of labour with fetal or maternal compromise and category three: poor progress of labour with no fetal or maternal compromise, planned CS with ruptured membranes, failed induction of labour, maternal medical conditions like pre-eclampsia is interpreted when there is evidence of maternal or fetal compromise which is not immediately life threatening. Category three (scheduled) includes (poor progress of labour with no fetal or maternal compromise, planned CS with ruptured membranes, failed induction of labour, maternal medical conditions like pre-eclampsia) emergency caesarean sections performed when there is no maternal or fetal concern, but early delivery is required. Category four are elective cases where delivery is done at the convenient time for the mother and the staff 1-2

The decision to delivery interval (DDI) is the duration in min from the time of decision making to delivery of the fetus by emergency caesarean section. A shorter DDI is required in most urgent situations for better maternal and fetal outcome. However, the 30-minute interval is not realistic especially in poor resource setting 3 as reported in several studies done in Ghana, Uganda and Tanzania where there was only 1.7% ,0.7 and 12% CS performed within 30 min respectively 4-6 . In addition, the studies conducted in Tanzania at Muhimbili National Hospital (MNH) and at a referral hospital in northern Tanzania reported that 0% and 12% caesarean delivery were performed within 30 min respectively 6-7.

It has been suggested that DDI should be less than 30 min for category one CS and 75 min for category two CS. This time limit will reduce both maternal and fetal complication 8. Several studies reported that in emergency CS, maternal and perinatal outcomes deteriorate when the DDI exceeds 75 min and may lead to poor outcome. 9-11 Categorization according to urgency in emergency CS improves communication among the service providers as well as reduces the morbidity to maternal and neonates 11. This study aimed to determine DDI for category one, two and three emergency CS and describe factors for delay at a tertiary National Hospital, Dar es salaam as a baseline for planned intervention following increased maternal and perinatal morbidity and mortality despite of high rate of caesarean section in our facility.

## Methods

This was a retrospective cross-sectional study involving case notes conducted at Muhimbili National Hospital (MNH), a tertiary referral National Hospital in Tanzania and a teaching hospital for Muhimbili University of Health and Allied Sciences located in Dar es Salaam region in Tanzania. MNH has an obstetric unit with three building and six wards: antenatal, postnatal, labour, maternal high dependency unit (HDU), maternal intensive care unit (ICU) and neonatal unit. The fourth building adjacent to operating theater will be constructed soon which will serve as obstetric triage for all patient admitted in obstetric unit. The obstetric theatre is in the third building located about 50 meters from the other two building and has four operating rooms for elective and emergency cesarean section as well as for gynecological procedures. The unit has around 750-800 deliveries per month of which about 60% are delivered by caesarean sections.

### Study participants

The recruitment of the study participant was done by systematic sampling after picking the first client from the register book in the labour ward who delivered by emergency caesarean section. Register book record all deliveries in the labour ward (spontaneous vaginal delivery and caesarean section delivery). Thereafter every second client who delivered by emergency CS in the register book was enrolled into the study after listing all women who delivered by emergency caesarean section. This sampling was done retrospectively for all emergency CS which was done from 15th September to 15th November, 2017.

The case notes of the sampled clients were traced and collected 24 hours after emergency CS where all recordon caesarean section was extracted and recorded in the questionnaire. The inclusion criteria were women who were admitted with fetal heart rate and gestation age of 34 to 42 completed weeks to avoid long waiting time for the women who were using dexamethasone to enhance lung maturity for gestation age below 34 weeks. Client case notes including pre-operative and anesthetic checklists were used to extract information as well as time when the decision to conduct emergency CS was made. Time to arrive in operating room and time of starting operation was extracted from pre- operative checklist and was used to calculate duration of staying in operating room before anesthesia induction. Anesthesia charts was used to calculate time when induction of anesthesia was initiated and time when the baby was delivered. DDI was defined as the time between decisions to conduct emergency CS to the actual time when the baby was delivered. The computed DDI was recorded in min as a continuous variable, then it was categorized into ≤30min, 31 to 75 min,76 to 120 min and ≥ 120 min.

There was no patient interview and confidentiality were assured and maintained as no names were used, each client was assigned a study identification/code number with a separate matching hospital file number for data referencing. The ethical clearance was granted by Senate Research and Publication Committee for Muhimbili University of Health and Allied Sciences (MUHAS). The permission to conduct the study was provided by the Executive Director, MNH.

### Data analysis

Descriptive statistics were summarized using frequency and proportions for categorical variables and measures of central tendency and dispersion for continuous variables using statistical package for social science (SPSS) version 23. A Chi square test was used to determine the associations between a set of variables and DDI during bivariate analysis. Odds ratio (OR) and 95% confidence interval for maternal and fetal outcomes associated with DDI were estimated using Logistic regression models. A P- value of less than 0.05 was considered statistically significant.

## Results

During the study period there were 1627 deliveries, 1036 (63.7%) underwent CS. Out of these 1036, 832 (80.3%) were emergency and 204 (19.7%) elective cases. Among 832 emergency CS, 779 (93.6%) women met inclusion criteria and 427 were included in the study. The minimum and maximum DDI for emergency CS were 21 and 521 min respectively.

Table 1 shows that more than half 280 (65.6%) delivered at term,64.4% were referred from other health facilities and 37% had age range of 25 to 29 years with mean age of 28.39 + 5.448. The mean gestation age at delivery was 37.56+2.267.

**Table 1:** Distribution of participants' demographic, obstetric and delivery characteristics(n=427)

Variables	Frequency n (%)
**Age**	
15 – 19	19 (4.4)
20 – 24	78 (18.3)
25 – 29	158 (37.0)
30 – 34	110 (25.8)
35 – 39	51 (11.9)
≥40	11 (2.6)
**Parity**	
1	141 (33.0)
2 – 4	280 (65.6)
≥5	6 (1.4)
**Marital status**	
Single	33 (7.7)
Married	367 (85.9)
Divorced/Widowed	1 (0.2)
Cohabiting	26 (6.1)
**Level of education**	
No formal education	22 (5.2)
Primary school level	195 (45.7)
Secondary school level	116 (27.2)
College/University level	94 (22.0)
**Occupation**	
Unemployed	146 (34.2)
Employed	76 (17.8)
Petty trader	201 (47.1)
Student	4 (0.9)
**Gestation age at delivery**	
34-36 weeks	148 (34.7)
37-42 weeks	279 (65.3)
**Admission status**	
Referred	275 (64.4)
Not referred	152 (35.6)
**Time of the day for surgery**	
0700 - 1859 HRS	192 (45.0)
1900 - 0659 HRS	235 (55.0)
**Type of anaesthesia**	
Spinal	396 (92.7)
General	20 (4.7)
Both	11 (2.6)

Only two (0.5%) of the emergency CS were performed within the recommended DDI of 30 min for category one and 13(14.8%) for category two (75 min) whereas category three it was 15(6.3%) at DDI of 75 min.

**Figure 2:**
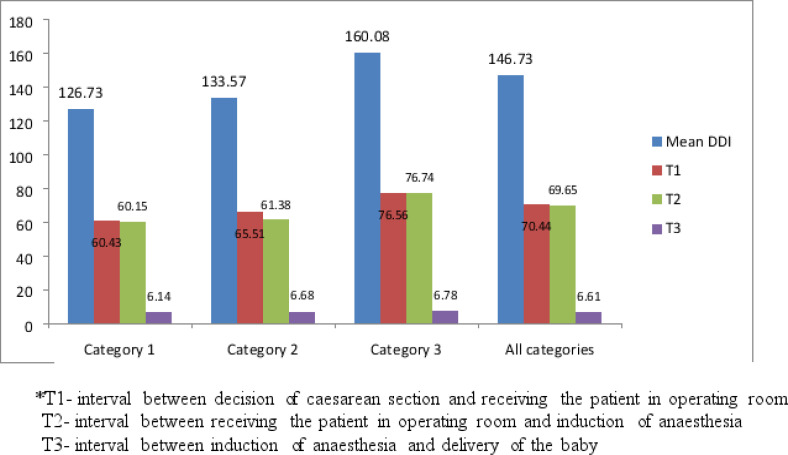
Time intervals at various levels from decision to delivery

Figure 2 above shows the mean time spent after decision time to the operating room was 70.44 min. In the receiving area in operating room, it took an average of 69.65 min before induction of anaesthesia.

Majority of maternal complication 58 (85.3%) occurred following prolonged DDI of more than 75 min as well as fetal complication such as low Apgar score ≤ 7, fresh still births, need for resuscitation and admission to NICU.

The adverse outcome in both maternal and fetal outcome were decreasing across the categories in relation to CS urgencies and it was clinically significant.

*a undischarged neonates at the time of data collection was included in the group of adverse neonatal outcome*

Adverse maternal and fetal outcome were more in DDI above 75 min with OR 1.2(0.49 to 2.83) and 1.7(0.91 to 3.38) times higher respectively than when the DDI was less than or equal to 75 min. However, this association was not statistically significant.

Having a lot of emergency surgery and poor communication between the team was documented as among the causes of delay in performing emergency cesarean section

## Discussion

This study found that the mean decision to delivery interval for category one was 126.73 min and only 0.5% of emergency CS were performed within recommended 30 min whereas the overall DDI was 146.73min. Patients spent considerable time during preparation for Caesarean delivery in the labour ward and in operation room reception before operation which were 70.44 and 69.65 min respectively. Mean DDI from this study is still far from the recommended 30 min interval for category one CS. The overall adverse maternal outcome was 7.5%, but when DDI was >75 min in

In category one there is an increase in maternal morbidity compared to ≤ 75 min (13.5%). The study found that there is progressive increase of adverse fetal outcome with increase in DDI. There was 1.7-fold increase in neonatal adverse outcome when the time was >75 min and this increased chances of having fetal complication compared to recommended time of ≤ 75 min. Operating room being busy and poor communication between the on-call teams was documented to be among the causes of delay in performing emergency cesarean section.

The DDI reported in this study was comparable to other studies which was more than 120 min 3-4;7. With respect to categories, the mean DDI were 126.73, 133.57 and 160.08 min for category one, two and three respectively. The decisions to delivery intervals were far from the recommended 30- and 75-min intervals for category one and two emergency Caesarean sections. To achieve the recommended DDI in a busy obstetric unit particularly in developing world is a challenge. There is a need to improve quality of the provided surgical intervention 12-13 in our units in particular communication between the labour and operating units so as to reduce the waiting time 14. Similar findings were reported in the studies done in Nigeria, Ghana and Uganda 4-5;15. However, in Europe and some Asian countries the mean DDI is reported to be short 16-17. The results from this study show an improvement of mean decision to delivery interval compared to previous study done about 10 years ago which was 200 min 7, however meeting the standard recommended time in this institution is beyond reach unless there is adjustment as it has been noted in several studies to be 75 min 18.

It is clear in this study that short interval between induction of anesthesia and delivery of the baby, this implies that the prolonged DDI was due to longer duration from decision for CS to induction of anesthesia. However, intervention for factors affecting DDI mentioned in this study is something to be studied in future in particular when the institution will be having a working obstetrics triage which is under construction 18-20. The short interval between induction of anesthesia and delivery of the baby could have been contributed by experience of both surgeon and anesthetist.

In the current study, category one CS was associated with poor maternal and neonatal outcome when DDI was > 75 min compared to category two and three respectively. There is an increase of morbidity to both maternal and fetal following prolonged DDI of > 75 min, the increase was 1.2 and 1.7 times for maternal and fetal respectively. More babies with low Apgar score of ≤7 at five min, admission to NICU as well as still birth and early neonatal death were noted in prolonged DDI of >75 min likewise the maternal complication. This finding is not far from what has been reported by Warren et al 21-23. The commonest maternal complication reported included haemorrhage, hysterectomy and maternal admission to ICU. These were increasing with Urgency of category of CS and DDI, similar observations have been reported in England, India and Nigeria^2^;^15^;^18^;^24^.

Adverse maternal and neonatal outcome were not significantly associated with prolonged DDI in some condition like hypertensive disease, prolonged labour, obstructed labour and fetal distress. Maternal resuscitation and correction of the morbid condition led to prolonged DDI at the same time reduced maternal and neonatal complication. Similar studies which were conducted in Nigeria, Ghana and Uganda found no significant association of DDI with maternal outcome 4-5;15. However, in other setting where DDI was found to be prolonged majority of women had higher incidence of adverse outcome 25. The studies done in India and Uganda showed that DDI of > 75 and 150 min respectively were significantly associated with adverse maternal outcome 18;26.

Being a retrospective study utilizing the available documented information in the case notes, few reason were documented indicating the reason why prolonged time in labour ward and operating room before anesthesia induction. The study could not utilize the pantograph information for the details on intrapartum care of the patient. Despite that, the recruitment of the patient was done systematically and involved all patients who were sent for emergency Caesarean section in our hospital giving a real picture of decision making in our labour ward.

## Conclusion

The mean DDI for category one, two and three were similar regardless of its urgency. Most of the delay was due to theater being busy, poor communication between the on-call team and delay in theater preparation. The mean DDI for CS at MNH is longer than the recommended time worldwide. The proportions of adverse maternal and fetal outcome increased when DDI was more than 75 min.

Triage in labour ward and in the operating room should be done regularly to identify client who need urgency delivery by CS. There is a need to consider 75 min as a recommended cut off point for prolonged DDI for category one and two in low resource setting.

## Figures and Tables

**Figure 1:**
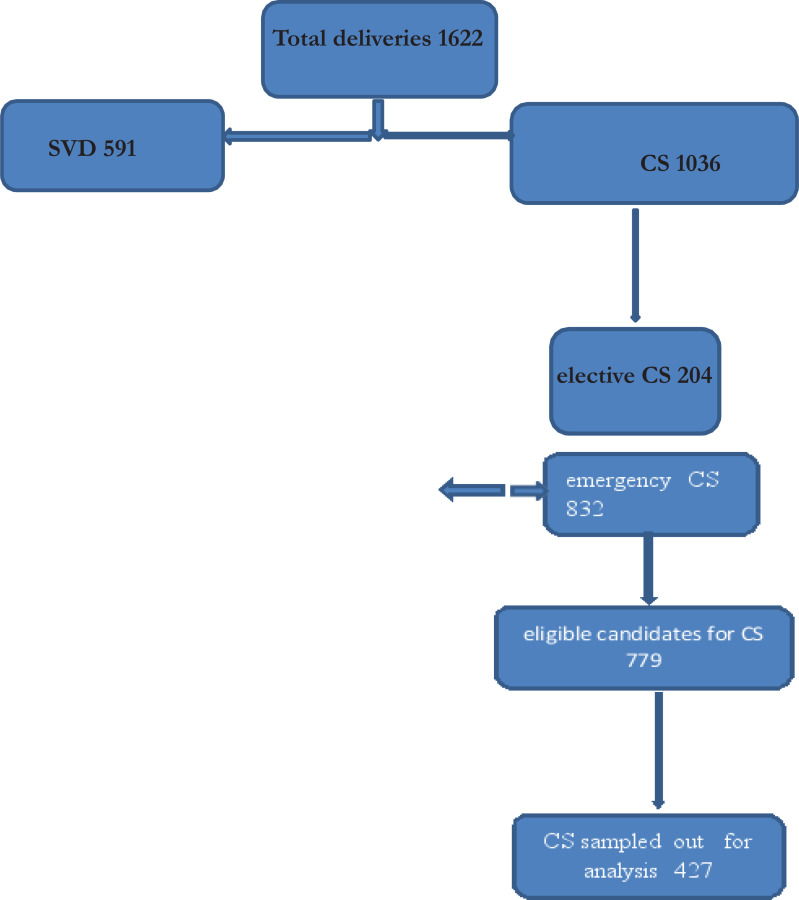
Flow Chart for Study Participants

**Table 2:** Mean decision to delivery interval (DDI) for different categories of urgency ofemergency Caesarean sections(n=427)

				DDI (min), Frequency n (%)		
Category	Mean DDI	<30	31 to 75	76 to 120	>120	Total
Category 1	126.73	2 (0.5)	24 (23.8)	34 (33.7)	41 (40.6)	101
Category 2	133.57	0 (0.0)	13 (14.8)	39 (44.3)	36 (40.9)	88
Category 3	160.08	0 (0.0)	15 (6.3)	68 (28.6)	155 (65.1)	238

*Mean DDI for emergency CS was 146.73 ± 75.79 min

**Table 3:** Mean decision to delivery interval (DDI) for different indications of emergency caesarean sections (n=427)

Indication	n (%)	Mean DDI + SD (min)
Fetal distress	63 (14.8)	131.7 + 69.1
Placenta praevia with haemorrhage	11 (2.6)	137.5 ± 89.3
Abruptio placentae	18 (4.2)	101.0 ± 62.0
Impending rupture of uterus	5 (1.2)	119.4 ± 82.4
Prolonged labour	42 (9.8)	123.6 ± 41.2
CPD	14 (3.3)	158.5 ± 58.7
Obstructed labour	32 (7.5)	119.8 ± 55.9
Previous uterine scar in labour	128(30.0)	146.8 ± 69.7
Failed VBAC	8 (1.9)	169.8 ± 141.4
Severe pre-eclampsia and eclampsia with unfavourable cervix	23 (5.4)	178 ± 78.5
Malpresentation	24 (5.6)	156.3 ± 58.2
Failed induction	38 (8.9)	176.4 ± 97.3
Multiple pregnancy with malpresentation	6 (1.4)	167.2 ± 16.3
Failed assisted vaginal delivery	1 (0.2)	268.0
Others	14 (3.3)	213.9 ± 119.1

Abruptio placentae had the mean DDI (101.0 ± 62.0 min).

**Table 4:** Adverse maternal and fetal outcomes in emergency caesarean sections (n=427)

	≤ 75 minn (%)	> 75 minn (%)
**Maternal outcome**		
Postpartum haemorrhage	2 (5.3)	36 (94.7)
Ruptured uterus	2 (66.7)	1 (33.3)
Extended uterine incision	3 (20.0)	12 (80.0)
Postpartum hysterectomy	1 (16.7)	5 (83.3)
Maternal admission to ICU	1 (20.0)	4 (80.0)
Maternal death	1 (100.0)	0 (0.0)
**Fetal**		
Apgar score less than 7	12 (12.1)	87 (87.9)
Need for resuscitation	11 (10.3)	96 (89.7)
Admission to NICU	7 (9.7)	65 (91.3)
Fresh still birth	6 (40.0)	9 (60.0)
Early neonatal death	1 (16.7)	5 (83.3)

*DDI of 75 min was used as a cut off interval to assess the effect of prolonged DDI on maternal and fetal outcomes in emergency CS

**Table 5:** Proportion of Maternal and neonatal outcome basing on the categories of urgency in CS N=427

Variables		Caesarean Category 1 n (%)	section Category 2 n (%)	categories Category 3 n (%)	Category 4 n (%)	Total n (%)	P-value
Maternal	Good outcome	1(50.0)	45(86.5)	131(92.9)	218(94.0)	395(92.5)	*0.152*
outcome	Adverse outcome	1(50.0)	7(13.5)	10(7.1)	14(6.0)	32(7.5)	

Fetal outcome	Good outcome	0(0)	38(73.1)	119(84.4)	205(88.4)	362(84.8)	*0.046*
	Adverse outcome	1(50)	8(15.4)	6(4.3)	11(4.7)	26(6.1)	
	Undischarged^α^ neonates	1(50)	6(11.5)	16(11.3)	16(6.9)	39(9.1)	

**Table 6:** Association of DDI with maternal and fetal outcomes in emergency CS (n=427)

	Maternal outcome OR 95% CI				Fetal outcome OR 95% CI			

DDI	Good outcome *n* (%)	Adverse outcome *n* (%)			Good outcome *n*(%)	Adverse outcome *n*(%)		
≤75 min	48 (88.8)	6 (11.2)	1		41(75.9)	13 (24.1)	1	
>75 min	319(85.5)	54 (14.5)	1.2	0.49 – 2.83	240 (64.3)	133 (35.7)	1.7	0.91 – 3.38

*Adverse maternal outcome means haemorrhage, hysterectomy and maternal admission to ICU

*Adverse fetal outcome means low Apgar score at 5 min of ≤ 7, need for neonatal resuscitation, admission to NICU, Fresh still birth and Early neonatal death and undischarged at the time of data collection

*Good maternal outcome means no complication was noted after emergency Caesarean section

*Good fetal outcome means Apgar score at 5 min of ≥ 7, no or minimal neonatal resuscitation and no admission to NICU.

**Table 7:** Documented cause of delays in performing emergency CS n=427

Variables	Number (%)
Theatre is being used by another emergency procedure	218(51.1)
Unavailability of Blood and blood products	19(4.4)
Unavailability of anesthetists	5(1.2)
Delay of surgeon	55(12.9)
Huge workload	62(14.5)
Difficult in achieving anesthesia	45(10.5)
Delay in theatre preparation	40(9.4)
Change of shift	22(5.2)
Lack of communication	56(13.1)
